# Low vaspin levels are related to endothelial dysfunction in patients with ankylosing spondylitis

**DOI:** 10.1590/1414-431X20165231

**Published:** 2016-07-04

**Authors:** H.H. Wang, Q.F. Wang

**Affiliations:** 1 Dongguan People's Hospital, Dongguan, Guangdong Province, China; 2 Department of Rheumatology, PAL Hospital, Beijing, China

**Keywords:** Vaspin, Endothelial function, Ankylosing spondylitis

## Abstract

Vaspin is a novel adipocytokine associated with glucose tolerance and chronic inflammation. Some studies reveal that vaspin may be involved in cardiovascular diseases. Our objective was to investigate the relationship between serum vaspin levels and endothelial function in patients with ankylosing spondylitis. One hundred and twenty patients with newly diagnosed ankylosing spondylitis and 100 healthy subjects were studied. Serum vaspin levels were measured with enzyme-linked immunosorbent assay. High resolution ultrasound was used to measure brachial artery diameter at rest, after reactive hyperemia (flow-mediated dilation, FMD) and after sublingual glyceryltrinitrate. Serum vaspin level in patients was 1.92±1.03 ng/mL, which was significantly lower than that in healthy subjects (2.88±0.81 ng/mL). By dividing the distribution of serum vaspin levels into quartiles, FMD levels increased gradually with the increase of serum vaspin levels in patients (P<0.01). Univariate analysis showed a correlation between vaspin and FMD (r=0.73, P=0.003), low-density lipoprotein cholesterol (r=-0.45, P=0.033), high-density lipoprotein cholesterol (r=0.63, P=0.025), fasting blood glucose (r=-0.79, P=0.006), triglycerides (TG) (r=-0.68, P=0.036), systolic blood pressure (r=-0.35, P=0.021), C-reactive protein (r=-0.67, P=0.011), homeostatic model assessment of insulin resistance (HOMA-IR) (r=-0.77, P=0.023) and erythrocyte sedimentation rate (r=-0.88, P=0.039) in patients. Multivariate analysis indicated that serum vaspin levels were independently associated with FMD, HOMA-IR and TG in patients. Our study found that serum vaspin levels were decreased in patients with ankylosing spondylitis and were associated with FMD levels. Vaspin may serve as an independent marker for detecting early stage atherosclerosis in patients with ankylosing spondylitis.

## Introduction

Vaspin is recognized as a novel adipokine in the modulation of adipocyte differentiation and glucose homeostasis (1,2). It has been proven to be a serine protease inhibitor family, which is associated with insulin resistance and metabolic syndrome (3). Furthermore, serum vaspin levels are associated with inflammation in rheumatoid arthritis (4,5). Recently, some studies have shown that vaspin has been linked to atherosclerosis and cardiovascular disease (6,7). Insulin resistance (IR) and chronic inflammation play an important role in the development of cardiovascular diseases (8,9). It has been assumed that vaspin serves as a potential insulin-sensitizing agent with anti-inflammatory effects and might act as a compensatory mechanism in the progression of atherosclerosis.

Endothelial dysfunction is an early event of atherosclerosis and plays a central role in the development and progression of cardiovascular diseases (10). Ankylosing spondylitis is associated with accelerated atherosclerosis and increased risk of metabolic syndrome (11-13). Clinical studies have shown that the impairment of flow-mediated dilation (FMD) is present in patients with ankylosing spondylitis (14-16). Sari et al., in a study where they excluded patients who had common cardiovascular risk factors, reported that ankylosing spondylitis had a role in the development of endothelial impairment (15). On the other hand, endothelial function of ankylosing spondylitis patients was impaired compared with healthy controls, and improved after tumor necrosis factor-alpha blockade (16).

Since no data is yet available on the relationship between serum vaspin levels and endothelial function in patients with ankylosing spondylitis, we tested the hypothesis that vaspin levels are associated with early stage atherosclerosis in patients with ankylosing spondylitis. The purpose of this study was to evaluate the relationship between vaspin and endothelial fucntion in patients with ankylosing spondylitis.

## Subjects and Methods

### Subjects

From January 2013 to September 2015, 120 newly diagnosed patients with ankylosing spondylitis and 100 age-matched healthy volunteers (controls) were enrolled in our study. The diagnosis of ankylosing spondylitis was based on the modified New York criteria (17). Exclusion criteria were set according to previous studies by our group (18,19). All participants who presented a history of congestive heart failure, myocardial infarction or angina were excluded. Besides, obese people (BMI >30 kg/m^2^), and people with malignant neoplasms, hypertension, renal or liver diseases were also excluded from our study. In addition, patients could not be taking any drugs, such as thyroxine, estrogen supplements, diuretics, antihypertensive or β-blockers, or lipid-lowering drugs. The study protocol was in agreement with the ethics guidelines of the committee at Dongguan People's Hospital, and was performed in accordance with the 1964 Declaration of Helsinki. Written consent or a thumb-print was required from all participants before screening.

### Methods

#### Biochemical measurements

The parameters were evaluated as described previously (18,19). Venous blood samples were drawn after a 12- to 14-h overnight fast. The serum vaspin levels were measured using a human vaspin sandwich enzyme-linked immunosorbent assay. Serum samples were diluted to 1/3 and measured in duplicate, and the results were averaged. The intra-assay coefficient of variation was 5-5.5%.

Total cholesterol (TC), triglycerides (TG), high-density lipoprotein cholesterol (HDL-C), and low-density lipoprotein cholesterol (LDL-C), were enzymatically determined. The fasting blood glucose (FBG) was analyzed by a glucose oxidase method. Levels of ultrasensitive C-reactive protein (CRP) levels were analyzed by immunoturbidimetric analysis. Erythrocyte sedimentation rate (ESR) and creatinine were determined using an automated analyzer (Sysmex XE5000, Japan). Coefficients of variation for these assays were 1-2% (TC, glucose, HDL-C), 1-2% (LDL-C, TG, CRP), and 1-3% (ESR).

#### Ultrasound examination of the brachial artery

Vascular examination of the brachial artery was performed noninvasively, as described previously (18,19). High resolution ultrasound was used to measure changes in arterial diameter in response to reactive hyperemia (with increased flow producing an endothelium-dependent stimulus to vasodilatation, FMD), and to glyceryltrinitrate (GTN-induced endothelium-independent arterial dilation; 128XP/10 with a 7.0-MHz linear array transducer; Acuson, USA). The intra- and inter-observer variability in our laboratory for repeated measurements of artery diameter is 0.09±0.10 and 0.08±0.13 mm, respectively.

### Statistical analysis

To compare numerical variables between two groups we used unpaired Student's *t*-test. The differences between categorical variables or nominal variables were analyzed with chi-square test. Univariate analysis was used for basic characteristics of participants expressed as categorical and continuous variables, including (FMD, vessel size, blood flow, GTN-induced endothelium-independent arterial dilation, LDL-C, HDL-C, TC, HOMA-IR, TG, CRP, diastolic and systolic blood pressure (DBP and SBP), FBG, age, ESR and BMI), while one-way analysis of variance (ANOVA) was used for categorical variables (smoking, family history and gender). Multiple linear regression analysis was performed to evaluate the association between vaspin and other independent variables. Pearson's correlation coefficients were calculated to determine the correlation between variables. Patient were stratified into four quartiles by vaspin levels. The between-group differences were evaluated by ANOVA. Significance was accepted at P<0.05. All calculations were performed by SPSS 13.0 (IBM, USA).

## Results

Table 1 shows the clinical characteristics of the control subjects and patients. Serum vaspin levels were significantly lower in patients with ankylosing spondylitis compared with the controls (1.92±1.03 *vs* 2.88±0.81 ng/mL, P<0.001). Ankylosing spondylitis subjects showed higher HOMA-IR, ESR, TG and CRP levels, compared with control group (P<0.01).

**Table:**
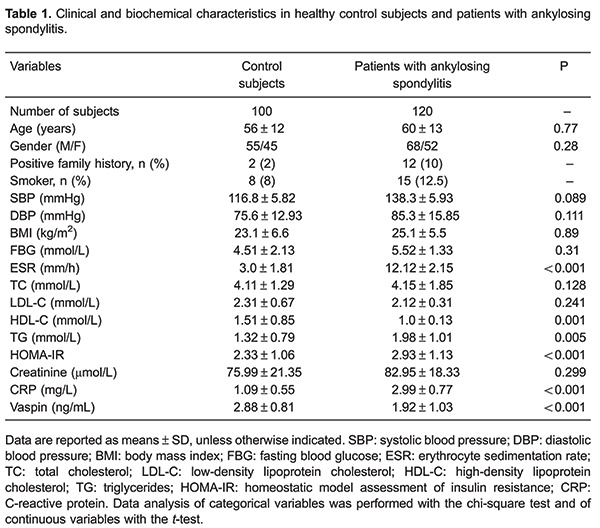

Patients were categorized into quartiles of serum vaspin levels (Table 2). These four groups did not show any difference in gender, age, BMI, positive family history, FBG, smoker, SBP, DBP, HDL-C, creatinine, baseline vessel, baseline flow and GTN-induced endothelium-independent arterial dilation (P>0.05). TG, CRP, HOMA-IR and ESR were gradually lower with higher serum vaspin levels (P<0.05). However, the FMD levels were gradually higher with higher serum vaspin levels (P<0.05). LDL-C and TC showed a tendency to be lower when serum vaspin levels were gradually higher (P>0.05).

**Table:**
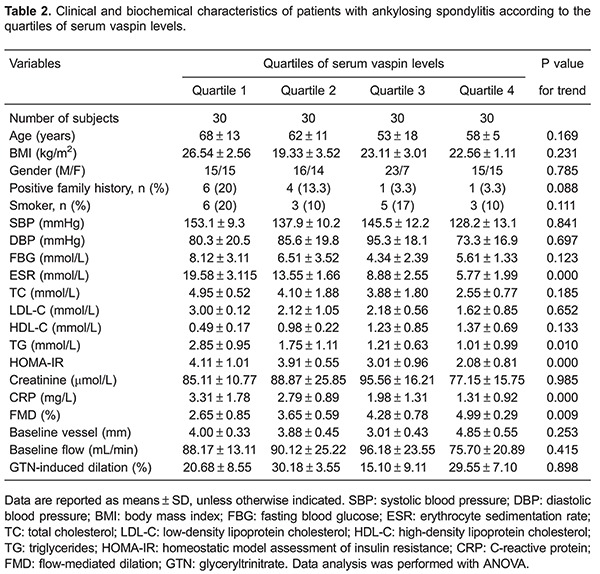

**Figure 1:**
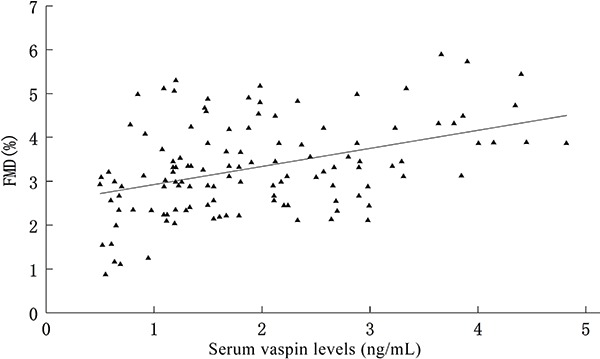
Correlation between serum vaspin levels and flow-mediated dilation (FMD) in patients with ankylosing spondylitis.

Univariate analysis between serum vaspin levels and variables such as FMD (r=0.73, P=0.003), LDL-C (r=−0.45, P=0.033), HDL-C (r=0.63, P=0.025), FBG (r=−0.79, P=0.006), TG (r=−0.68, P=0.036), SBP (r=-0.35, P=0.021), CRP (r=−0.67, P=0.011), HOMA-IR (r=−0.77, P=0.023) and ESR (r=−0.88, P=0.039) showed significant correlations (P<0.05). By multivariate regression analysis, FMD, LDL-C, HDL-C, FBG, TG, SBP, CRP, HOMA-IR, and ESR were independently associated to serum vaspin levels in patients with ankylosing spondylitis. The significant determinants of serum vaspin levels were FMD, HOMA-IR and TG. Pearson's analysis showed a significant association between serum vaspin levels and FMD levels (P<0.05; Figure 1).

## Discussion

Direct evidence of the role of vaspin in endothelial function is lacking, especially in patients with ankylosing spondylitis. We found that serum vaspin levels are related with endothelial function, and significantly decreased in patients with ankylosing spondylitis, compared to controls. Our study indicated that vaspin might be employed as a novel prognostic marker in patients with ankylosing spondylitis.

Vaspin, containing 415 amino acids secreted by visceral adipose tissue, was reported to modulate different functions as a ligand for a cell-surface receptor complex (1). Nakatsuka et al. have demonstrated that vaspin-transgenic mice are protected against diet-induced obesity, glucose tolerance impairment and fatty liver (20). Administration of recombinant vaspin improves glucose intolerance, and insulin sensitivity in diet-induced obese mice (21). Vaspin alleviates the dysfunction of endothelial progenitor cells induced by high glucose (22). In addition, a previous study reported that vaspin inhibited the progression of atherosclerotic plaques in apoE^−/−^ mice by inhibiting endoplasmic reticulum stress-induced macrophage apoptosis (23). Increased vaspin expression may be an intrinsic compensatory mechanism in adipose tissue as a response to decreased insulin sensitivity or impairment of glucose metabolism (24). Vaspin levels were found to have an inverse relationship with the risk of cardiovascular events, suggesting a protective role of vaspin in the pathophysiology of coronary atherosclerosis (25). More recently, clinical studies have shown that vaspin was associated with insulin resistance, chronic inflammation and might be involved in the development of metabolic diseases (3). Our study reports the new adipokine vaspin in ankylosing spondylitis, and it shows that vaspin is significantly lower in ankylosing spondylitis compared with healthy controls. The mechanism related with lower vaspin levels was not investigated in our study. Since vaspin also exerts anti-inflammatory properties, like the suppression of TNF-alpha (26), it would be conceivable that lower vaspin levels might be a consequence of the increase of pro-inflammatory cytokines in ankylosing spondylitis. In the present study, vaspin was correlated with CRP and ESR, which are in agreement with this hypothesis.

Much controversy exists about the relationship of vaspin with different chronic inflammatory diseases. Morisaki et al. (27) have shown that vaspin plasma levels are elevated in patients with ulcerative colitis and increase further after remission induction. The study by Ozgen et al. (5) shows an important association between vaspin and chronic inflammatory diseases. They reported that vaspin levels are higher in rheumatoid arthritis and may be involved in the regulation of inflammatory responses in inflammatory diseases. Moreover, in contrast to rheumatoid arthritis, vaspin levels decline in active Behcet's disease. The above studies suggest that different chronic inflammatory diseases exert different influences on vaspin levels. Thus, the relationship between vaspin and different chronic inflammatory diseases needs to be confirmed by further studies with larger number of subjects.

Numerous studies have reported on the association of vaspin with cardiovascular diseases (6,7). It has been reported that vaspin concentrations are associated with coronary atherosclerosis (7). Esaki et al. (6) published an observational study among 201 subjects from the general population in Japan, where they retrospectively measured vaspin levels. They found an independent association between serum vaspin levels and intima-media thickness. Moreover, previous works have suggested that low vaspin emerged as an important factor for coronary artery disease (28). Endothelial dysfunction is one of the first signs of atherosclerosis and cardiovascular diseases. Brachial FMD has been a widely used noninvasive method for the assessment of vascular endothelial function. Many clinical studies showed that ankylosing spondylitis is associated with vascular endothelial dysfunction, which leads to accelerated atherosclerosis. However, few studies have evaluated the association of vaspin with endothelial function in patients with ankylosing spondylitis. The present study found that vaspin was associated with endothelial function, in which serum vaspin levels in patients increased gradually as FMD levels increased from quartile 1 to quartile 4 (Table 2).

Metabolic syndromes are considered diseases with common traits that can increase the risk of cardiovascular disease (29). Ankylosing spondylitis is a chronic inflammatory disease associated with a metabolic syndrome (30). In current clinical practice, we found that serum vaspin levels were negatively correlated with TG, CRP and HOMA-IR, which is in agreement with a previous study (31). In addition, LDL-C and TC levels decreased when serum vaspin levels increased gradually.

The effects of vaspin on endothelial function as well as the regulatory mechanisms in ankylosing spondylitis are poorly understood. FMD is a proposed indicator of nitric oxide-bioavailability and vascular function (10). Vaspin increases nitric oxide bioavailability through the reduction of asymmetric dimethylarginine in vascular endothelial cells (32). The study by Sun et al. (22) indicated a novel effect of vaspin to regulate eNOS expression and function in endothelial progenitor cells via a PI3K/Akt/eNOS pathway, and vaspin may have a protective effect in patients with diabetes in preventing the occurrence of vascular complications. Further studies are needed to explain the mechanism by which vaspin relates with endothelial function in ankylosing spondylitis.

The most significant limitation of this study is the small sample size and we can not exclude the effect of other adipokines such as irisin, chemerin and adiponectin. Besides, the corresponding levels of cell-surface receptor complex was not evaluated. Another limitation is that intra-observer variation was not assessed in this study.

In conclusion, this study showed that serum vaspin levels were decreased in patients with ankylosing spondylitis, and were significantly associated with endothelial function. The mechanism for the relationship between vaspin and endothelial function in ankylosing spondylitis needs to be explored in the future.
